# Aortic Root Dilatation Is Attenuated with Diabetes but Is Not Associated with Renal Progression in Chronic Kidney Disease

**DOI:** 10.3390/jpm11100972

**Published:** 2021-09-28

**Authors:** Pei-Yu Wu, Jiun-Chi Huang, Yi-Hsueh Liu, Ho-Ming Su, Szu-Chia Chen, Yi-Wen Chiu, Jer-Ming Chang

**Affiliations:** 1 Division of Nephrology, Department of Internal Medicine, Kaohsiung Medical University Hospital, Kaohsiung Medical University, Kaohsiung 807, Taiwan; wpuw17@gmail.com (P.-Y.W.); karajan77@gmail.com (J.-C.H.); chiuyiwen@gmail.com (Y.-W.C.); jemich@kmu.edu.tw (J.-M.C.); 2 Department of Internal Medicine, Kaohsiung Municipal Siaogang Hospital, Kaohsiung Medical University, Kaohsiung 812, Taiwan; Liuboy17@gmail.com (Y.-H.L.); cobeshm@seed.net.tw (H.-M.S.); 3 Faculty of Medicine, College of Medicine, Kaohsiung Medical University, Kaohsiung 807, Taiwan; 4 Division of Cardiology, Department of Internal Medicine, Kaohsiung Medical University Hospital, Kaohsiung Medical University, Kaohsiung 807, Taiwan

**Keywords:** chronic kidney disease, aortic root dilatation, diabetes mellitus, left atrial dimension, eGFR slope, left ventricular mass index

## Abstract

Patients with chronic kidney disease (CKD) often have cardiac functional and structural abnormalities which can lead to adverse cardiovascular outcomes. In this study, we investigated associations between diabetes mellitus (DM) and cardiac functional and structural parameters in patients with CKD focusing on aortic root diameter (ARD). We also investigated associations of renal outcomes with DM and cardiac functional and structural characteristics. We enrolled 419 patients with CKD stage 3–5 were enrolled. ARD was normalized to body surface area (BSA) (ARD/BSA), and the rate of decline in renal function was assessed by the estimated glomerular filtration rate (eGFR) slope (mL/min/1.73 m^2^/year). ARD/BSA ≥2.1 cm/m^2^ in men or ≥2.2 cm/m^2^ in women was defined as indicating aortic root dilatation. The patients with DM had lower ARD/BSA, higher left atrial dimension (LAD), lower left ventricular ejection fraction, lower ratio of peak early transmitral filling wave velocity to peak late transmitral filling wave velocity, and higher left ventricular relative wall thickness, than those without DM. After multivariable analysis, DM (vs. non-DM; coefficient β, −0.060; *p* = 0.018) was significantly associated with low ARD/BSA. Significantly fewer patients with DM had aortic root dilatation compared to those without DM (14.3% vs. 23.1%, *p* = 0.022). In the patients with DM, there were significant associations between a high left ventricular mass index (LVMI) (per 1 g/m^2^, β, −0.016; *p* = 0.040) and high LAD (per 1 cm; β, −1.965; *p* < 0.001) with a low eGFR slope. However, other parameters, including ARD/BSA, were not associated with eGFR slope. Furthermore, there were no associations between eGFR slope and any of the echocardiographic parameters in the patients without DM. Aortic root dilatation was attenuated in the patients with DM, but it was not associated with a decline in renal function. However, high LAD and LVMI were associated with rapid renal function decline in the CKD patients with DM.

## 1. Introduction

Patients with chronic kidney disease (CKD) are at high risk of cardiovascular (CV) morbidity and mortality due to many factors [1]. Traditional risk factors include hypertension, diabetes mellitus (DM) and hyperlipidemia in addition to CKD itself [2]. The pathogenesis of CV disease in CKD may be associated with endothelial dysfunction, inflammation, oxidative stress and uremic toxins [2]. In addition, CV disease in CKD has been associated with cardiac remodeling involving both functional and structural abnormalities. Left ventricular hypertrophy (LVH) and diastolic dysfunction are common in patients with CKD. LVH occurs in the early stage of CKD, with an increasing incidence in the advanced stages [3,4]. A low left ventricular ejection fraction (LVEF) has been associated with mortality in patients with CKD, and worsening LVEF and left ventricular end-systolic volume (LVESV) when transitioning from CKD to dialysis have also been associated with post-dialysis mortality [5,6]. In addition, the progression of diastolic dysfunction to symptomatic heart failure has been associated with CKD, and CKD has also been associated with aortic valve calcification and arterial stenosis [7,8]. Evaluating cardiac function is important in patients with CKD, however the use of contrast imaging for this purpose is limited by the risk of contrast-induced nephrotoxicity in these patients. Consequently, cardiac echography has become the most commonly used tool to evaluate cardiac function in patients with CKD due to its non-invasiveness and ease of use.

Increased aortic root diameter (ARD) has been reported to be a predictor of thoracic aortic aneurysm rupture [9], and aortic root dilatation has been associated with a high risk of heart failure, CV and all-cause mortality even without aortic aneurysm [10,11,12]. In addition, aortic root dilatation has been reported to be a predictor of left ventricular diastolic dysfunction, and the association between aortic root dilatation and LVH has been demonstrated to be a stronger predictor of CV prognosis than LVH alone [12,13]. Masugata et al. reported an inverse association between estimated glomerular filtration rate (eGFR) and aortic root dilatation in patients with hypertension [14], and that DM was inversely correlated with ARD in patients undergoing peritoneal dialysis [15]. However, few studies have investigated correlations among DM, aortic root dilatation and renal function decline in patients with CKD.

In this study, we investigated relationships between DM and cardiac functional and structural parameters, with a focus on ARD, in patients with CKD. We also investigated associations of renal outcomes, assessed using eGFR slope, with DM and cardiac functional and structural characteristics.

## 2. Methods and Patients

### 2.1. Study Design and Patients

This study was conducted at Kaohsiung Medical University Hospital (KMUH), Taiwan, from May 2006 to January 2010. The Institutional Review Board of KMUH approved this study, which followed the appropriate guidelines. We enrolled 505 patients with CKD stage 3–5. None of the enrolled patients had initiated dialysis. CKD was defined as the presence of kidney disease for three months or longer. The stages of CKD were defined in accordance with the National Kidney Foundation-Kidney Disease Outcomes Quality Initiative (K/DOQI) guidelines [16] as follows (all eGFR values in mL/min/1.73 m^2^): stage 3, eGFR 30–59; stage 4, eGFR 15–29; and stage 5, eGFR < 15. To ensure sufficient data to be able to analyze renal function changes, patients with fewer than three follow-up eGFR measurements (*n* = 64), and those with less than six months of data (*n* = 22) during the follow-up period were excluded. In total, we included 419 patients (256 males and 163 females; mean age 65.9 ± 12.3 years), all of whom provided written informed consent before enrollment. 

### 2.2. Evaluation of Cardiac Structure and Function

The echocardiographic examinations were conducted by two experienced cardiologists who were unaware of the patients’ data using an ultrasound machine (VIVID 7, General Electric Medical Systems, Horten, Norway). Two-dimensional and M-mode echocardiographic measurements were performed with the patients in a left decubitus position. ARD was normalized to body surface area (BSA) (ARD/BSA). Aortic root dilatation was defined as ARD/BSA ≥2.1 cm/m^2^ in men or ≥2.2 cm/m^2^ in women [12]. LVEF was used to assess left ventricular systolic function. Peak early transmitral filling wave velocity (E), peak late transmitral filling wave velocity (A), and E/A ratio, LAD, LVESV, left ventricular internal diameter in diastole (LVIDd), left ventricular posterior wall thickness in diastole (LVPWTd), left ventricular end-diastolic volume (LVEDV), and interventricular septal wall thickness in diastole (IVSTd) were measured. Observed left ventricular mass (LVM) was calculated using the modified Devereux method: observed LVM = 1.04 × [(IVSTd + LVIDd + LVPWTd)^3^ − LVIDd^3^] − 13.6 g [17]. LVMI was calculated as LVM/BSA.

### 2.3. Medical, Demographic and Laboratory Data

Data on age, sex, smoking status, baseline information on hypertension, cerebrovascular disease, DM and coronary artery disease were collected by chart review. Patients with a history of cerebral bleeding and infarction were defined as having cerebrovascular disease, and those with a history of ischemic electrocardiogram change, old myocardial infarction, angina, angioplasty or coronary bypass surgery were defined as having coronary artery disease. Blood pressure (both systolic and diastolic) measurements were recorded. Fasting blood samples were collected from each patient within 1 month of enrollment, and levels of triglycerides, glucose, total cholesterol, hemoglobin and calcium-phosphorous product were measured at out laboratory. Serum creatinine levels were determined using the kinetic Jaffé method with isotope-dilution mass spectrometry [18]. The CKD Epidemiology Collaboration (CKD-EPI) Study Equation was used to calculate eGFR [19]. The dipstick method was used to evaluate proteinuria (Hema-Combistix, Bayer Diagnostics), and a positive test result was defined as a score of 1+ or more. Urine samples were obtained within 1 month of enrollment. The patients’ medical records were searched for information on prescriptions including angiotensin II receptor blockers (ARBs), angiotensin converting enzyme inhibitors (ACEIs) and statins.

### 2.4. Rate of Renal Function Decline

The eGFR slope was used to assess the rate of decline in renal function measured in mL/min/1.73 m^2^/year. The slope was calculated as the regression coefficient between time and a minimum of three eGFR measurements recorded after enrollment.

### 2.5. Statistical Analysis

Data are presented as percentages, means ± standard deviations, and medians (25th–75th percentiles), and means ± standard errors of the mean for eGFR slope. One-way analysis of variance (ANOVA) was used for multi-group comparisons followed by post hoc adjustments with Bonferroni correction. The independent t-test was used to examine differences between groups for continuous variables, and the chi-square test was used for categorical variables. linear regression analysis was performed to examine associations between the echocardiographic parameters with ARD/BSA and eGFR slope. A *p* value of less than 0.05 was considered to indicate a significant difference. SPSS version 26.0 for Windows was used for all statistical analyses (SPSS Inc., Chicago, IL, USA).

## 3. Results 

### 3.1. Comparisons of the Clinical Characteristics between ARD/BSA Tertiles

The clinical characteristics were compared between ARD/BSA tertiles (Table 1). The patients in ARD/BSA tertile 3 were older, predominantly male, and had lower triglycerides and total cholesterol than those in ARD/BSA tertile 1. For the echocardiographic parameters, the patients in ARD/BSA tertile 3 had a lower LAD and higher LVMI and LVESV than those in ARD/BSA tertile 1.

### 3.2. Comparisons of Echocardiographic Parameters between the Patients with and without DM

The echocardiographic parameters were compared between the patients with and without DM (Table 2). The patients with DM had a lower ARD/BSA, LVEF and E/A, and higher LAD and LVRWT than those without DM.

### 3.3. Determinants of ARD/BSA

Table 3 shows the risk factors for ARD/BSA in multivariable stepwise linear regression analysis. Old age (per 1 year; unstandardized coefficient β, 0.005; *p* < 0.001), male (vs. female; β, −0.062; *p* = 0.019), low triglycerides (log per 1 mg/dL; β, −0.159; *p* = 0.002), and low baseline eGFR (per 1 mL/min/1.73m^2^; β, −0.002; *p* = 0.007) were significantly associated with a large ARD/BSA. In addition, DM (vs. non-DM; β, −0.060; *p* = 0.018) was negatively significantly associated with ARD/BSA.

**Figure 1:**
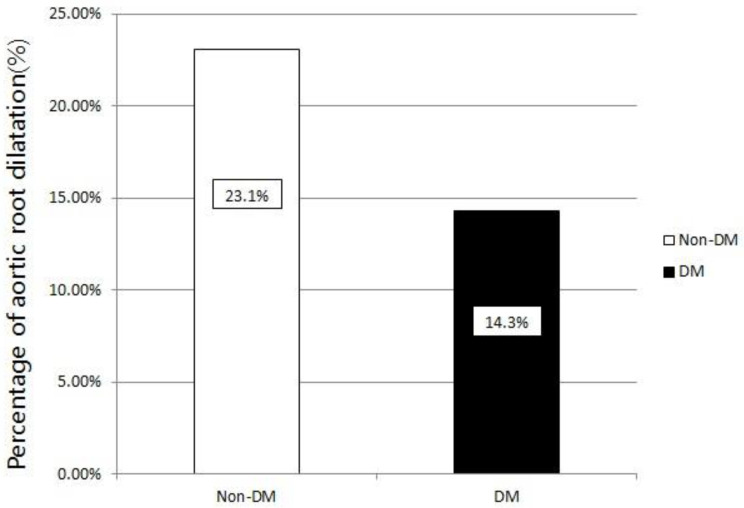
Percentage of aortic root dilatation in patients with or without diabetes. Aortic root dilatation was defined as aortic root diameter (ARD)/body surface area (BSA) ≥ 2.1 cm/m^2^ in women or 2.2 cm/m^2^ in men. In patients with diabetes, the percentage of aortic root diameter dilatation was significantly lower, when compared with those without diabetes (14.3% vs. 23.1%, *p* = 0.022). DM, diabetes mellitus.

Figure 1 illustrates the percentage of aortic root dilatation in the study patients with and without DM. Significantly fewer patients with DM had aortic root dilatation compared to those without DM (14.3% vs. 23.1%, *p* = 0.022). 

### 3.4. Associations between eGFR Slope and Echocardiographic Parameters

Associations between eGFR slope and the echocardiographic parameters after multiple adjustments for age, sex, smoking history, cerebrovascular disease, log triglycerides, total cholesterol, systolic blood pressure > 140 mmHg, hemoglobin, hypertension, coronary artery disease, fasting glucose, eGFR, calcium-phosphorous product, proteinuria and the use of ACEIs and/or ARBs are shown in Table 4. In the patients with DM, high LAD (per 1 cm; β, −1.965; *p* < 0.001) and high LVMI (per 1 g/m^2^, β, −0.016; *p* = 0.040) were significantly associated with low eGFR slope. However, other parameters, including ARD/BSA, were not associated with eGFR slope. Furthermore, in the patients without DM, none of the echocardiographic parameters were associated with eGFR slope.

Analysis of the effects of interactions between echocardiographic parameters and DM on eGFR slope is also shown in Table 4. The interaction between LAD and DM (*p* = 0.010) was statistically significant. However, interactions of the other parameters and DM did not achieve significance.

## 4. Discussion

In this study, we examined associations among echocardiographic parameters and DM, and further evaluated the associations with renal outcomes in patients with CKD. Our results showed a significant association between DM and low ARD/BSA. In addition, in the patients with DM, high LAD and high LVMI were significantly associated with a low eGFR slope, however ARD/BSA was not associated with eGFR slope. Furthermore, in the patients without DM, none of the echocardiographic parameters were associated with eGFR slope.

The first important findings of this study are that compared to the patients without DM, those with DM had lower ARD/BSA, and that significantly fewer of the patients with DM had aortic root dilatation. A previous meta-analysis of 8 studies reported that the prevalence of aortic root dilatation in patients with hypertension was around 10%, and that is was even higher in male and elderly patients [20], which is consistent with the study by Mule et al [14]. In our study, the prevalence of aortic root dilatation was higher in the patients without DM than in those with DM. In addition, old age and male sex were correlated with high ARD/BSA, which is consistent with hypertensive patients [20]. Ye et al. evaluated the relationship between DM and ARD in patients with end-stage renal disease (ESRD) receiving peritoneal dialysis, and found an inverse association between DM and ARD. Moreover, DM was independently correlated with a low prevalence of aortic root dilatation [15]. Cuspid et al. investigated the relationships between aortic root size and different markers of cardiac and extracardiac target organ damage in patients with hypertension, and reported a prevalence of DM of 5.2%, and that DM was not correlated with ARD [21]. However, they did not report information on renal function or the prevalence of CKD [21]. Aortic root dilatation has been linked with thoracic aortic aneurysm rupture, and DM has been reported to have a protective effect against thoracic and abdominal aortic aneurysm [22,23]. Several mechanisms influencing the relationship between DM and aortic aneurysm may explain the lower ARD in DM patients. Changes in the extracellular matrix (ECM) and loss of vascular smooth muscle cells (VSMCs) have been shown to contribute to thoracic aortic aneurysm formation [24]. The transforming growth factor-beta (TGF-β) pathway involves ECM formation and has been linked to thoracic aortic aneurysm formation, and it is up-regulated in DM [25,26]. Metalloproteinases (MMPs) degrade ECM protein and are also regulated by DM. In addition, hyperglycemia has been shown to increase the expression of plasminogen activator inhibitor-1 and reduce plasma levels of MMP-9 in mice, and this effect may attenuate aortic aneurysm diameter [27]. Furthermore, the TGF-β pathway up-regulates and decreases the MMP-9 expression involved in changes in the ECM, and this may contribute to the protective effect of DM against aortic aneurysm formation. Krüppel-like factor 4 controls VMSC phenotypic transition in atherosclerosis plaque formation and is involved in aortic aneurysm formation, and it has been shown to be down-regulated by hyperglycemia in VMSCs [24,28,29]. The TGF-β pathway regulates VSMC differentiation and protects the thoracic aorta from intramural hematoma formation, medial and adventitial thickening. This prevents the pathology of thoracic aortic aneurysm, and up-regulation of the TGF-β pathway in DM may explain the influence on aortic aneurysm [30,31]. The influence of DM in changes in the ECM and VMSCs in thoracic aneurysms may explain the role of DM on ARD in patients with CKD.

We also found an association between low eGFR and high ARD/BSA, but that high ARD/BSA was not associated with rapid renal function decline. Only a few studies have investigated the association between renal function and ARD, and the relationship between the rate in the decline of eGFR and ARD is unclear. A previous study on hypertension reported an inverse association between eGFR and ARD [14], and that albuminuria was not correlated with aortic root size [21]. Vascular calcification occurs in patients with CKD, and it is associated with arterial stiffness. Some studies have reported associations between arterial stiffness and cardiac remodeling in patients with CKD and renal outcomes, and the results of our study may share the same mechanism [32]. A low eGFR has been associated with higher carotid artery internal diameter and increased carotid circumferential wall stress in CKD patients. This has been linked to an increase in the internal diameter of the carotid artery, which in turn has been independently associated with a decline in GFR [33,34]. The Chronic Renal Insufficiency Cohort (CRIC) study enrolled 2564 CKD participants of whom one-half had DM, and demonstrated that aortic pulse wave velocity (as a marker of arterial stiffness) was significantly associated with reduced renal function and DM [35]. 

Another important finding of the present study is that high LAD and LVMI were associated with a rapid decline in renal function in the patients with DM. The interactions among cardiac dysfunction, DM and CKD are complex, and hemodynamic changes, hypoperfusion, oxidative stress, endothelial dysfunction, cytokines and neurohormonal abnormalities all play roles in organ damage associated with cardiac dysfunction and CKD progression [36]. In our previous studies, we found that LVH was associated with the progression of patients with CKD stage 3–5 to ESRD, and that high LAD and LVMI were correlated with poor renal outcomes [37,38]. Fibroblast growth factor 23 (FGF-23) is a hormone which regulates mineral metabolism, and it is elevated in patients with CKD. The FGF-23/Klotho protein axis may explain the association between LVH with renal function in CKD patients with DM. In addition, FGF-23 has been associated with insulin resistant, and a recent review paper discussed the role of FGF-23 as a predictor of CV events [39]. One study of 55 patients with macroalbuminuric diabetic nephropathy found that FGF-23 was associated with CKD progression, including doubling of creatinine and progression to dialysis [40]. Another meta-analysis study of 34 studies which included 9 non-dialysis CKD populations found that a higher FGF-23 level increased the risk of myocardial infarction, heart failure and stroke [41]. In addition, Gutierrez et al. studied 162 non-dialysis CKD patients, and demonstrated that FGF-23 was independently correlated with LVMI and LVH, which is consistent with a study by Mirza et al. in elderly patients [42,43]. Several experimental studies have also demonstrated that FGF-23 regulates cardiac remodeling with LVH via the activation of cardiac phospholipase/calcineurin/nuclear factor signaling in activated T-cells through FGF receptor 4 [44,45]. However, the mechanism has yet to be fully elucidated, and further studies are needed to clarify the role of FGF-23 in the association between LVMI and renal outcomes in CKD patients with DM. Left atrial enlargement, which is a long-term consequence of remodeling and affects left ventricular filling pressure can be used to assess left ventricular diastolic function to help in the diagnosis of heart failure, and in a study of 305 DM patients without CV disease, left atrial enlargement was associated with CV morbidity and mortality [46]. Hyperglycemia-induced interstitial fibrosis and insulin resistance can both increase blood pressure, and this may worsen left atrial remodeling and function [47]. Several previous studies have reported that an enlarged left atrium is a predictor of CV mortality, atrial fibrillation, congestive heart failure, stroke and hypertension, and this could be useful clinically [48,49,50,51]. In addition, in Furukawa et al.’s study of 140 patients with CKD stage 4–5, the authors demonstrated an independent association between left atrial volume index and the time to initiating dialysis [52]. In the current study of CKD patients with DM, we demonstrated an independent association between a high LAD and rapid renal function decline. This suggests that a high LAD may be associated with a high volume status and left ventricular filling pressure. Consequently, these patients may have higher renal efferent pressure and lower renal blood flow, which could then result in a progressive renal function decline [53]. 

There were several limitations to this study. First, the number of serum creatinine measurements and the intervals between them differed from patient to patient, and thus the eGFR slope also differed. However, only patients who were followed for at least 3 months and had at least three eGFR measurements were included in this study, which may have increased the reliability of the eGFR slope findings. Second, this study was conducted at a single center, and this the result may not be generalizable to all patients with CKD. Third, albuminuria is an early marker of diabetic nephropathy and an important factor influencing DM outcomes, however we did not quantify albuminuria in this study. In addition, the effect of volume overload could not be evaluated as we did not assess volume status. Finally, we lacked data on lipid-lowering agent history, and these medications undoubtedly influence the values of lipid profiles. Therefore, we could not exclude the impact of such medications on our results.

In conclusion, aortic root dilation was attenuated in the CKD patients with DM in this study, but it was not associated with renal outcomes. In addition, high LAD and LVMI were associated with rapid decline in renal function in the patients with DM. 

## Figures and Tables

**Table 1:** Comparison of clinical characteristics between the groups according to ARD/BSA tertile.

Characteristics	Tertile 1 (*n* = 139)	Tertile 2 (*n* = 140)	Tertile 3 (*n* = 140)	*p*
Age (year)	62.2 ± 13.6	66.0 ± 11.5 *	69.6 ± 10.6 *^,†^	<0.001
Male sex (%)	52.5	64.3	66.4	0.037
Smoking (%)	30.2	27.1	34.3	0.431
DM (%)	62.6	55.7	51.4	0.166
Hypertension (%)	84.2	85.0	80.0	0.492
Coronary artery disease (%)	10.1	13.6	10.0	0.559
Cerebrovascular disease (%)	16.5	12.9	16.4	0.624
Systolic blood pressure (mmHg)	140.6 ± 21.0	140.8 ± 19.5	142.8 ± 23.0	0.653
Diastolic blood pressure (mmHg)	79.0 ± 12.2	80.1 ± 13.8	79.4 ± 12.5	0.761
Laboratory parameters				
Fasting glucose (mg/dL)	130.8 ± 60.0	121.6 ± 58.8	126.7 ± 54.3	0.411
Triglyceride (mg/dL)	152 (99–239)	151 (99.75–200.25)	128 (80–178) *	0.002
Total cholesterol (mg/dL)	200.2 ± 44.5	196.5 ± 46.1	187.0 ± 46.9 *	0.046
Hemoglobin (g/dL)	11.8 ± 2.3	11.6 ± 2.3	11.7 ± 2.3	0.691
Baseline eGFR (mL/min/1.73 m^2^)	27.0 ± 14.0	23.2 ± 13.3	25.1 ± 14.5	0.076
Calcium-phosphorous product (mg^2^/dL^2^)	38.5 ± 8.2	37.0 ± 7.9	38.4 ± 9.4	0.252
Proteinuria (%)	64.7	67.1	65.5	0.911
Medications				
ACEI and/or ARB (%)	77.8	75.0	71.9	0.540
Statins (%)	28.1	23.5	27.3	0.653
Echocardiographic parameters				
ARD/BSA (cm/m^2^)	1.68 ± 0.11	1.94 ± 0.06 *	2.23 ± 0.17 *^,†^	<0.001
LAD (cm)	3.90 ± 0.61	3.72 ± 0.61	3.59 ± 0.64 *	<0.001
LVRWT (%)	44.61 ± 11.99	43.71 ± 12.33	44.24 ± 14.70	0.878
LVMI (g/m^2^)	132.32 ± 42.19	137.91 ± 43.63	146.35 ± 51.54 *	0.038
LVEDV (mL)	111.32 ± 31.42	113.43 ± 33.59	119.06 ± 47.64	0.219
LVESV (mL)	33.72 ± 16.99	36.16 ± 17.72	40.85 ± 33.90 *	0.044
LVEF (%)	70.44 ± 9.13	69.03 ± 8.83	68.00 ± 11.70	0.124
E/A	0.89 ± 0.31	0.81 ± 0.26	0.85 ± 0.46	0.208
Renal outcome				
eGFR slope (mL/min/1.73 m^2^/yr)	−2.24 ± 0.35	−2.11 ± 0.45	−1.50 ± 0.37	0.368

Abbreviations. ARD, aortic root diameter; BSA, body surface area; DM, diabetes mellitus; eGFR, estimated glomerular filtration rate; ACEI, angiotensin-converting enzyme inhibitor; ARB, angiotensin II receptor blocker; LAD, left atrial dimension; LVRWT, left ventricular relative wall thickness; LVMI, left ventricular mass index; LVEDV, left ventricular end-diastolic volume; LVESV, left ventricular end-systolic volume; LVEF, left ventricular ejection fraction; E, peak early transmitral filling wave velocity; A, peak late transmitral filling wave velocity. * *p* < 0.05 compared to tertile 1; ^†^ *p* < 0.05 compared to tertile 2.

**Table 2:** Comparison of echocardiographic parameters in study patients without or with DM.

Echocardiographic Parameters	without DM (*n* = 182)	with DM (*n* = 237)	*p*
ARD/BSA (cm/m^2^)	1.99 ± 0.27	1.92 ± 0.23	0.009
LAD (cm)	3.59 ± 0.63	3.85 ± 0.61	<0.001
LVRWT (%)	42.32 ± 11.79	45.62 ± 16.47	0.017
LVMI (g/m^2^)	136.06 ± 46.3	141.05 ± 46.17	0.274
LVEDV (mL)	114.74 ± 34.39	114.51 ± 41.12	0.952
LVESV (mL)	35.45 ± 20.36	38.05 ± 26.93	0.261
LVEF (%)	70.25 ± 8.94	68.30 ± 10.69	0.043
E/A	0.92 ± 0.43	0.80 ± 0.27	0.001

Abbreviations are the same as in Table 1.

**Table 3:** Determinants of ARD/BSA using multivariable stepwise linear analysis.

Parameters	ARD/BSA	
	Unstandardized Coefficient β (95% CI)	*p*
Age (per 1 year)	0.005 (0.003, 0.007)	<0.001
Sex (male vs. female)	0.062 (0.010, 0.113)	0.019
DM	−0.060 (−0.109, −0.010)	0.018
Triglyceride (log per 1 mg/dL)	−0.159 (−0.261, −0.057)	0.002
Baseline eGFR (per 1 mL/min/1.73 m^2^)	−0.002 (−0.004, −0.001)	0.007

Values expressed as unstandardized coefficient β and 95% confidence interval (CI). Abbreviations are the same as in Table 1. Multivariable model: adjusted for age, sex, smoking history, hypertension, coronary artery disease, cerebrovascular disease, systolic blood pressure > 140 mmHg, fasting glucose, log triglyceride, total cholesterol, hemoglobin, eGFR, calcium-phosphorous product, proteinuria, ACEI and/or ARB and statins.

**Table 4:** Relation of echocardiographic parameters to eGFR slope using multivariable linear analysis.

Echocardiographic Parameters	without DM (*n* = 182)			with DM (*n* = 237)			
	Multivariable			Multivariable			
	Unstandardized Coefficient β	95% CI	*p*	Unstandardized Coefficient β	95% CI	*p*	Interaction *p*
ARD/BSA (per 1 cm/m^2^)	1.281	−0.782, 3.344	0.222	0.471	−2.419, 3.362	0.748	0.929
LAD (per 1 cm)	−0.040	−0.864, 0.784	0.924	−1.965	−3.006, −0.925	<0.001	0.010
LVRWT (per 1%)	0.011	−0.037, 0.058	0.664	−0.007	−0.045, 0.031	0.730	0.265
LVMI (per 1 g/m^2^)	−0.006	−0.018, 0.006	0.343	−0.016	−0.032, -0.001	0.040	0.396
LVEDV (per 1 mL)	0.001	−0.016, 0.018	0.870	−0.016	−0.032, 0.001	0.062	0.508
LVESV (per 1 mL)	0.003	−0.024, 0.031	0.814	−0.022	−0.047, 0.003	0.082	0.351
LVEF (per 1%)	−0.008	−0.067, 0.051	0.801	0.026	−0.039, 0.091	0.424	0.285
E/A (per 1)	−0.052	−1.324, 1.219	0.935	−0.333	−2.991, 2.325	0.805	0.198

Values expressed as unstandardized coefficient β and 95% confidence interval (CI). Abbreviations are the same as in Table 1. Multivariable model: adjusted for age, sex, smoking history, hypertension, coronary artery disease, cerebrovascular disease, systolic blood pressure >140 mmHg, fasting glucose, log triglyceride, total cholesterol, hemoglobin, eGFR, calcium-phosphorous product, proteinuria, and ACEI and/or ARB.

## Data Availability

Data may be available upon request to interested researchers. Please send data requests to: Szu-Chia Chen. Division of Nephrology, Department of Internal Medicine, Kaohsiung Medical University Hospital, Kaohsiung Medical University.

## References

[1] Van der Velde M., Matsushita K., Coresh J., Astor B.C., Woodward M., Levey A., de Jong P., Gansevoort R.T., Chronic Kidney Disease Prognosis Consortium (2011). Lower estimated glomerular filtration rate and higher albuminuria are associated with all-cause and cardiovascular mortality. A collaborative meta-analysis of high-risk population cohorts. Kidney Int..

[2] Gansevoort R.T., Correa-Rotter R., Hemmelgarn B.R., Jafar T.H., Heerspink H.J., Mann J.F., Matsushita K., Wen C.P. (2013). Chronic kidney disease and cardiovascular risk: Epidemiology, mechanisms, and prevention. Lancet.

[3] Pluta A., Strozecki P., Krintus M., Odrowaz-Sypniewska G., Manitius J. (2015). Left ventricular remodeling and arterial remodeling in patients with chronic kidney disease stage 1–3. Ren. Fail..

[4] Park M., Hsu C.Y., Li Y., Mishra R.K., Keane M., Rosas S.E., Dries D., Xie D., Chen J., He J. (2012). Associations between kidney function and subclinical cardiac abnormalities in CKD. J. Am. Soc. Nephrol..

[5] Wu I.W., Hung M.J., Chen Y.C., Hsu H.J., Cherng W.J., Chang C.J., Wu M.S. (2010). Ventricular function and all-cause mortality in chronic kidney disease patients with angiographic coronary artery disease. J. Nephrol..

[6] Bansal N., Roy J., Chen H.Y., Deo R., Dobre M., Fischer M.J., Foster E., Go A.S., He J., Keane M.G. (2018). Evolution of Echocardiographic Measures of Cardiac Disease From CKD to ESRD and Risk of All-Cause Mortality: Findings From the CRIC Study. Am. J. Kidney Dis..

[7] Vogel M.W., Slusser J.P., Hodge D.O., Chen H.H. (2012). The natural history of preclinical diastolic dysfunction: A population-based study. Circ. Heart Fail..

[8] Patel K.K., Shah S.Y., Arrigain S., Jolly S., Schold J.D., Navaneethan S.D., Griffin B.P., Nally J.V., Desai M.Y. (2019). Characteristics and Outcomes of Patients With Aortic Stenosis and Chronic Kidney Disease. J. Am. Heart Assoc..

[9] Davies R.R., Gallo A., Coady M.A., Tellides G., Botta D.M., Burke B., Coe M.P., Kopf G.S., Elefteriades J.A. (2006). Novel measurement of relative aortic size predicts rupture of thoracic aortic aneurysms. Ann. Thorac. Surg..

[10] Gardin J.M., Arnold A.M., Polak J., Jackson S., Smith V., Gottdiener J. (2006). Usefulness of aortic root dimension in persons > or = 65 years of age in predicting heart failure, stroke, cardiovascular mortality, all-cause mortality and acute myocardial infarction (from the Cardiovascular Health Study). Am. J. Cardiol..

[11] Lai C.L., Chien K.L., Hsu H.C., Su T.C., Chen M.F., Lee Y.T. (2010). Aortic root dimension as an independent predictor for all-cause death in adults <65 years of age (from the Chin-Shan Community Cardiovascular Cohort Study). Echocardiography.

[12] Cuspidi C., Facchetti R., Bombelli M., Re A., Cairoa M., Sala C., Tadic M., Grassi G., Mancia G. (2014). Aortic root diameter and risk of cardiovascular events in a general population: Data from the PAMELA study. J. Hypertens..

[13] Masugata H., Senda S., Murao K., Okuyama H., Inukai M., Hosomi N., Iwado Y., Noma T., Kohno M., Himoto T. (2011). Aortic root dilatation as a marker of subclinical left ventricular diastolic dysfunction in patients with cardiovascular risk factors. J. Int. Med. Res..

[14] Mule G., Nardi E., Morreale M., D’Amico S., Foraci A.C., Nardi C., Geraci G., Cerasola G., Cottone S. (2016). Relationship between aortic root size and glomerular filtration rate in hypertensive patients. J. Hypertens..

[15] Ye M., Zhang J., Li J., Liu Y., He W., Lin H., Fan R., Li C., Li W., Liu D. (2019). Diabetes attenuated age-related aortic root dilatation in end-stage renal disease patients receiving peritoneal dialysis. J. Diabetes Investig..

[16] Initiative K.D.O.Q. (2002). K/DOQI clinical practice guidelines for chronic kidney disease: Evaluation, classification, and stratification. Am. J. Kidney Dis..

[17] Devereux R.B., Alonso D.R., Lutas E.M., Gottlieb G.J., Campo E., Sachs I., Reichek N. (1986). Echocardiographic assessment of left ventricular hypertrophy: Comparison to necropsy findings. Am. J. Cardiol..

[18] Vickery S., Stevens P.E., Dalton R.N., van Lente F., Lamb E.J. (2006). Does the ID-MS traceable MDRD equation work and is it suitable for use with compensated Jaffe and enzymatic creatinine assays?. Nephrol. Dial. Transplant..

[19] Levey A.S., Stevens L.A., Schmid C.H., Zhang Y.L., Castro A.F., Feldman H.I., Kusek J.W., Eggers P., van Lente F., Greene T. (2009). A new equation to estimate glomerular filtration rate. Ann. Intern. Med..

[20] Covella M., Milan A., Totaro S., Cuspidi C., Re A., Rabbia F., Veglio F. (2014). Echocardiographic aortic root dilatation in hypertensive patients: A systematic review and meta-analysis. J. Hypertens..

[21] Cuspidi C., Meani S., Fusi V., Valerio C., Sala C., Zanchetti A. (2006). Prevalence and correlates of aortic root dilatation in patients with essential hypertension: Relationship with cardiac and extracardiac target organ damage. J. Hypertens..

[22] Takagi H., Umemoto T., Group A. (2017). Negative Association of Diabetes With Thoracic Aortic Dissection and Aneurysm. Angiology.

[23] Prakash S.K., Pedroza C., Khalil Y.A., Milewicz D.M. (2012). Diabetes and reduced risk for thoracic aortic aneurysms and dissections: A nationwide case-control study. J. Am. Heart Assoc..

[24] Raffort J., Lareyre F., Clement M., Hassen-Khodja R., Chinetti G., Mallat Z. (2018). Diabetes and aortic aneurysm: Current state of the art. Cardiovasc. Res..

[25] Mallat Z., Ait-Oufella H., Tedgui A. (2017). The Pathogenic Transforming Growth Factor-beta Overdrive Hypothesis in Aortic Aneurysms and Dissections: A Mirage?. Circ. Res..

[26] Li J., Huynh P., Dai A., Wu T., Tu Y., Chow B., Kiriazis H., Du X.J., Bach L.A., Wilkinson-Berka J.L. (2018). Diabetes Reduces Severity of Aortic Aneurysms Depending on the Presence of Cell Division Autoantigen 1 (CDA1). Diabetes.

[27] Dua M.M., Miyama N., Azuma J., Schultz G.M., Sho M., Morser J., Dalman R.L. (2010). Hyperglycemia modulates plasminogen activator inhibitor-1 expression and aortic diameter in experimental aortic aneurysm disease. Surgery.

[28] Shankman L.S., Gomez D., Cherepanova O.A., Salmon M., Alencar G.F., Haskins R.M., Swiatlowska P., Newman A.A., Greene E.S., Straub A.C. (2015). KLF4-dependent phenotypic modulation of smooth muscle cells has a key role in atherosclerotic plaque pathogenesis. Nat. Med..

[29] Hien T.T., Garcia-Vaz E., Stenkula K.G., Sjogren J., Nilsson J., Gomez M.F., Albinsson S. (2018). MicroRNA-dependent regulation of KLF4 by glucose in vascular smooth muscle. J. Cell Physiol..

[30] Angelov S.N., Hu J.H., Wei H., Airhart N., Shi M., Dichek D.A. (2017). TGF-beta (Transforming Growth Factor-beta) Signaling Protects the Thoracic and Abdominal Aorta From Angiotensin II-Induced Pathology by Distinct Mechanisms. Arterioscler. Thromb. Vasc. Biol..

[31] Kanzaki T., Shiina R., Saito Y., Zardi L., Morisaki N. (1997). Transforming growth factor-beta receptor and fibronectin expressions in aortic smooth muscle cells in diabetic rats. Diabetologia.

[32] Briet M., Boutouyrie P., Laurent S., London G.M. (2012). Arterial stiffness and pulse pressure in CKD and ESRD. Kidney Int..

[33] Briet M., Bozec E., Laurent S., Fassot C., London G.M., Jacquot C., Froissart M., Houillier P., Boutouyrie P. (2006). Arterial stiffness and enlargement in mild-to-moderate chronic kidney disease. Kidney Int..

[34] Briet M., Collin C., Karras A., Laurent S., Bozec E., Jacquot C., Stengel B., Houillier P., Froissart M., Boutouyrie P. (2011). Arterial remodeling associates with CKD progression. J. Am. Soc. Nephrol..

[35] Townsend R.R., Wimmer N.J., Chirinos J.A., Parsa A., Weir M., Perumal K., Lash J.P., Chen J., Steigerwalt S.P., Flack J. (2010). Aortic PWV in chronic kidney disease: A CRIC ancillary study. Am. J. Hypertens..

[36] Ronco C., Bellasi A., Di Lullo L. (2018). Cardiorenal Syndrome: An Overview. Adv. Chronic Kidney Dis..

[37] Chen S.C., Su H.M., Hung C.C., Chang J.M., Liu W.C., Tsai J.C., Lin M.Y., Hwang S.J., Chen H.C. (2011). Echocardiographic parameters are independently associated with rate of renal function decline and progression to dialysis in patients with chronic kidney disease. Clin. J. Am. Soc. Nephrol..

[38] Huang T.H., Chiu H., Wu P.Y., Huang J.C., Lin M.Y., Chen S.C., Chang J.M. (2021). The association of echocardiographic parameters on renal outcomes in chronic kidney disease. Ren. Fail..

[39] Berezin A.E., Berezin A.A. (2019). Impaired function of fibroblast growth factor 23/Klotho protein axis in prediabetes and diabetes mellitus: Promising predictor of cardiovascular risk. Diabetes Metab. Syndr..

[40] Titan S.M., Zatz R., Graciolli F.G., dos Reis L.M., Barros R.T., Jorgetti V., Moyses R.M. (2011). FGF-23 as a predictor of renal outcome in diabetic nephropathy. Clin. J. Am. Soc. Nephrol..

[41] Marthi A., Donovan K., Haynes R., Wheeler D.C., Baigent C., Rooney C.M., Landray M.J., Moe S.M., Yang J., Holland L. (2018). Fibroblast Growth Factor-23 and Risks of Cardiovascular and Noncardiovascular Diseases: A Meta-Analysis. J. Am. Soc. Nephrol..

[42] Gutierrez O.M., Januzzi J.L., Isakova T., Laliberte K., Smith K., Collerone G., Sarwar A., Hoffmann U., Coglianese E., Christenson R. (2009). Fibroblast growth factor 23 and left ventricular hypertrophy in chronic kidney disease. Circulation.

[43] Mirza M.A., Larsson A., Melhus H., Lind L., Larsson T.E. (2009). Serum intact FGF23 associate with left ventricular mass, hypertrophy and geometry in an elderly population. Atherosclerosis.

[44] Grabner A., Amaral A.P., Schramm K., Singh S., Sloan A., Yanucil C., Li J., Shehadeh L.A., Hare J.M., David V. (2015). Activation of Cardiac Fibroblast Growth Factor Receptor 4 Causes Left Ventricular Hypertrophy. Cell Metab..

[45] Leifheit-Nestler M., Richter B., Basaran M., Nespor J., Vogt I., Alesutan I., Voelkl J., Lang F., Heineke J., Krick S. (2018). Impact of Altered Mineral Metabolism on Pathological Cardiac Remodeling in Elevated Fibroblast Growth Factor 23. Front. Endocrinol..

[46] Poulsen M.K., Dahl J.S., Henriksen J.E., Hey T.M., Hoilund-Carlsen P.F., Beck-Nielsen H., Moller J.E. (2013). Left atrial volume index: Relation to long-term clinical outcome in type 2 diabetes. J. Am. Coll. Cardiol..

[47] Tadic M., Cuspidi C. (2021). Left atrial function in diabetes: Does it help?. Acta Diabetol..

[48] Abhayaratna W.P., Seward J.B., Appleton C.P., Douglas P.S., Oh J.K., Tajik A.J., Tsang T.S. (2006). Left atrial size: Physiologic determinants and clinical applications. J. Am. Coll. Cardiol..

[49] Todaro M.C., Choudhuri I., Belohlavek M., Jahangir A., Carerj S., Oreto L., Khandheria B.K. (2012). New echocardiographic techniques for evaluation of left atrial mechanics. Eur. Heart J. Cardiovasc. Imaging.

[50] Tsang T.S., Barnes M.E., Gersh B.J., Bailey K.R., Seward J.B. (2002). Left atrial volume as a morphophysiologic expression of left ventricular diastolic dysfunction and relation to cardiovascular risk burden. Am. J. Cardiol..

[51] Kizer J.R., Bella J.N., Palmieri V., Liu J.E., Best L.G., Lee E.T., Roman M.J., Devereux R.B. (2006). Left atrial diameter as an independent predictor of first clinical cardiovascular events in middle-aged and elderly adults: The Strong Heart Study (SHS). Am. Heart J..

[52] Furukawa M., Io H., Tanimoto M., Hagiwara S., Horikoshi S., Tomino Y. (2011). Predictive Factors Associated with the Period of Time before Initiation of Hemodialysis in CKD Stages 4 and 5. Nephron Clin. Pract..

[53] Bock J.S., Gottlieb S.S. (2010). Cardiorenal syndrome: New perspectives. Circulation.

